# Hypoxia and Mitochondrial Dysfunction in Pregnancy Complications

**DOI:** 10.3390/antiox10030405

**Published:** 2021-03-08

**Authors:** Xiang-Qun Hu, Lubo Zhang

**Affiliations:** Lawrence D. Longo, MD Center for Perinatal Biology, Department of Basic Sciences, Loma Linda University School of Medicine, Loma Linda, CA 92350, USA

**Keywords:** preeclampsia, fetal growth restriction, placenta, mitochondria, reactive oxygen species, oxidative stress, therapy

## Abstract

Hypoxia is a common and severe stress to an organism’s homeostatic mechanisms, and hypoxia during gestation is associated with significantly increased incidence of maternal complications of preeclampsia, adversely impacting on the fetal development and subsequent risk for cardiovascular and metabolic disease. Human and animal studies have revealed a causative role of increased uterine vascular resistance and placental hypoxia in preeclampsia and fetal/intrauterine growth restriction (FGR/IUGR) associated with gestational hypoxia. Gestational hypoxia has a major effect on mitochondria of uteroplacental cells to overproduce reactive oxygen species (ROS), leading to oxidative stress. Excess mitochondrial ROS in turn cause uteroplacental dysfunction by damaging cellular macromolecules, which underlies the pathogenesis of preeclampsia and FGR. In this article, we review the current understanding of hypoxia-induced mitochondrial ROS and their role in placental dysfunction and the pathogenesis of pregnancy complications. In addition, therapeutic approaches selectively targeting mitochondrial ROS in the placental cells are discussed.

## 1. Introduction

Preeclampsia and fetal growth restriction (FGR) are two most common pregnancy complications worldwide, affecting 5–10% of pregnancy [1,2,3]. According to the International Society for the Study of Hypertension in Pregnancy (ISSHP) [4], preeclampsia is defined as gestational hypertension with systolic blood pressure (BP) ≥ 140 and/or diastolic BP ≥ 90 mm Hg at or after 20 weeks’ gestation, accompanied by one or more of the following conditions: (1) proteinuria, (2) other maternal organ dysfunction such as acute kidney injury, liver dysfunction, neurological complications and hematological complications; and (3) uteroplacental dysfunction such as FGR, abnormal umbilical artery Doppler wave form analysis, or stillbirth. FGR refers the failure of a fetus to reach its genetic/biological growth potential and is clinically defined as an ultrasonographic estimated fetal weight or abdominal circumference below the 10th percentile for gestational age by the American College of Obstetricians and Gynecologists and Society for Maternal-Fetal Medicine [2,5]. Preeclampsia is heterogeneous, exhibiting in the early onset (premature delivery prior to 34 weeks’ gestation) and late-onset forms (delivery at or after 34 weeks’ gestation). Early onset preeclampsia is frequently associated with FGR [6]. Preeclampsia and FGR are major causes of maternal and perinatal morbidity and mortality [7,8]. Moreover, they also predispose for various diseases in later life of the mother and offspring, including cardiovascular disease, metabolic syndrome, and neurologic impairment [8,9,10,11].

It is now recognized that both preeclampsia and FGR originate from the placenta due to uteroplacental dysfunction conferred by gestational hypoxia [1,12,13,14,15]. Gestational hypoxia is associated with overproduction in reactive oxygen species (ROS) in the placenta, leading to oxidative stress [12,16,17,18]. ROS can be beneficial or deleterious, depending on their levels in cells. At low levels, ROS function as signaling molecules in regulating a variety of cellular processes [19]. However, due to their highly reactivity, the overwhelming accumulation of ROS may indiscriminately damage cellular macromolecules such as lipids, proteins and DNAs and consequently impairs cellular functions. Oxidative stress has been implicated in many human diseases including hypertension [20,21]. Not surprisingly, uteroplacental tissues/cells of preeclampsia and FGR display a heightened level of oxidative stress [22], which plays a central role in the pathogenesis of both complications [18,23]. Mitochondria consume most cellular oxygen (O_2_) to produce ATP to sustain cellular functions and a significant portion of ROS is also generated during the oxidation-phosphorylation coupling. Mitochondria are severely impacted by reduced O_2_ availability as hypoxia alters mitochondrial structure/dynamics and electron transfer complexes and enhances production of ROS [24,25]. It appears that mitochondria are the predominant sources to generate ROS in the placenta under both physiological and pathophysiological conditions [22,26]. Here, we review current knowledge of placental mitochondrial ROS in the pathogenesis of preeclampsia and FGR. In addition, we also discuss the potential therapeutic strategy by targeting mitochondrial ROS in the management of these two pregnancy complications.

## 2. Hypoxia, Hypoxia Inducible Factors (HIFs) and Preeclampsia/FGR

### 2.1. Maladaptation of Uteroplacental Circulation in Preeclampsia/FGR

Uterine vessels undergo substantially structural and functional adaptation during pregnancy. Structural changes of uterine vessels involve an increase arterial lumen with the remodeling of spiral arteries [27], whereas functional changes of uterine arteries implicate modulating ion channel expression/activity to lowering intrinsic myogenic tone [14]. The uterine vascular adaptation along with the formation of the placenta establishes the low-resistance and high-flow uteroplacental circulation, resulting in a dramatic increase in uteroplacental perfusion to meet the requirement of placental and fetal growth/development. However, uteroplacental hemodynamic is altered in preeclampsia and FGR, displaying increased uteroplacental vascular resistance and reduced uteroplacental perfusion [27,28,29,30,31,32]. Impaired trophoblast invasion and subsequent deficient spiral arteries are prominent features of preeclampsia and FGR [33]. As expected, inhibition of trophoblast invasion in rat pregnancy results in uteroplacental hypoperfusion [34].

### 2.2. Hypoxia and Hypoxia Inducible Factors (HIFs)

Oxygen is essential to the survival of the most eukaryotic organisms. In general, hypoxia constitutes a severe stress to cells. Mounting evidence suggests that hypoxia is a key player in the pathogenesis of preeclampsia and FGR [14,15,35]. Hypoxia-inducible factors (HIFs) are key molecules that regulate cellular responses to hypoxia and play important roles in both physiological and pathophysiological contexts. HIFs are transcription factors consisting of HIF-α subunit (HIF-1α or HIF-2α) and HIF-1β. Both α and β subunits are constitutively expressed and only the α subunit is regulated by O_2_ levels. Under normoxia, the α subunit is hydroxylated by prolyl hydroxylase domain proteins (PHDs), which use O_2_ and α-ketoglutarate as substrates. The hydroxylated α subunit is then recognized and polyubiquitylated by the von Hippel-Lindau (VHL) protein that serves as an E3 ubiquitin ligase, followed by proteasomal degradation. However, under hypoxic conditions, PHDs are enzymatically inactive and the α subunit are no longer hydroxylated. Thus, the α subunit is stabilized and translocated to the nucleus, where the α subunit dimers with HIF-1β and activates expression of target genes, playing critical roles in mediating cellular responses to hypoxia.

### 2.3. Hypoxia, Hypoxia to Normoxia Transition and Normal Pregnancy

Intriguingly, hypoxia is essential for development including embryonic and placental development [36,37,38]. Measured at weeks 7–10 in the first trimester, the partial O_2_ pressure is ~20 mm Hg in the placenta and 60 mm Hg in the decidua [39]. The embryonic and placental development thus occurs under a hypoxic environment. Hypoxia in this period is not considered to be pathological as low O_2_ promotes trophoblast proliferation and angiogenesis/vasculogenesis in the placenta [40,41]. At weeks 11–16, placental O_2_ tension reaches ~60 mm Hg, approaching the O_2_ level in the decidua (~70 mm Hg) [39]. The rise in O_2_ is believed to trigger trophoblast to differentiate [40], promoting the transition of trophoblasts from the proliferative to an invasive phenotype to complete spiral artery remodeling. The establishment of the uteroplacental circulation allows to adequately supply O_2_ and nutrients to the placenta to meet the metabolic and biosynthetic demands for the rapid growth/development of the placenta and the fetus in the second and third trimesters.

### 2.4. Hypoxia and Preeclampsia/FGR

The expression of HIF-1α in the placenta occurs concurrently with placental O_2_ tension, displaying high expression in the first trimester and declining starting from week 9 of gestation [42,43]. Inactivation of HIF-1α in maternal tissues at E8.5 leads to reduced trophoblast proliferation and increased apoptosis in mouse placentas [44]. Most importantly, the temporal expression pattern of HIF-1α in trophoblasts is key to the shift from proliferation to invasiveness. Antisense inhibition of HIF-1α expression triggers a switch of trophoblasts from proliferative to invasive phenotype [42]. Failure to complete this transition would compromise spiral artery modeling. Placental hypoxia apparently persists beyond the first trimester in preeclampsia and FGR as HIF-1α and HIF-2α expression in the placenta keeps elevated throughout pregnancy [45,46,47,48,49]. Further evidence to support the pivotal role of hypoxia in the pathogenesis of preeclampsia and FGR comes from studies in which humans and animals experience hypobaric and/or normobaric hypoxia and from in or ex vivo studies using trophoblasts and placental explants. Pregnancy at high altitude is associated with ~3-fold increases in the incidence of preeclampsia and FGR [50,51,52] and overexpression of placental HIF-1α [53]. Pregnant ewes exposed to hypobaric hypoxia at high altitude have elevated blood pressure and uterine vascular resistance and reduced birthweight of newborn lambs [54,55]. Additionally, numerous animal studies demonstrated that chronic normobaric hypoxia during pregnancy increases placental HIF expression, inhibits trophoblast invasion, and promotes preeclampsia-like symptoms and FGR [56,57,58,59,60,61,62,63]. In vitro studies also demonstrated that hypoxia boosts HIFs expression in trophoblast cell lines [64,65]. Furthermore, the global gene expression displayed a strikingly similar pattern in placentas from preeclampsia and high-altitude pregnancy and ex vivo hypoxia-treated placental explants [66]. Pregnant mice with global overexpression of HIF-1α display phenotypes of preeclampsia and FGR, showing elevated blood pressure, proteinuria and reduced fetal weight [67]. Similar findings and impaired spiral artery remodeling are also observed with prolonged expression of trophoblast-specific HIF-1α [68]. In response to hypoxia, the induction of HIF-1α and HIF-2α also stimulates the production soluble Fms-like tyrosine kinase-1 (sFlt-1) in trophoblasts [64,69]. sFlt-1, an anti-angiogenic factor, is increased in circulation in preeclampsia and FGR [70,71,72]. It is believed that sFlt-1 contributes to the pathogenesis of preeclampsia by prompting endothelial dysfunction, disrupting angiogenesis, and impairing trophoblast invasion [70,73,74,75,76]. Low O_2_ tension is an inducer of angiogenesis. HIF-1α is a key regulator of placental vascular development. Genetic deletion of HIF-1α or HIF-2α results in defective placental vascularization [77]. In contrast, high altitude pregnancy increases HIF-1α expression in the placenta, which is associated with greater vascularity [78].

The functional adaptation of uterine arteries also contributes to the marked increase in uterine blood flow in pregnancy. Large-conductance Ca^2+^-activated K^+^ (BK_Ca_) channels and ryanodine receptors in uterine arteries appear to be major determinants in initiating the functional adaptation in pregnancy [79,80,81,82,83]. The coupling of ryanodine receptor-mediated Ca^2+^ sparks to BK_Ca_ channel-mediated spontaneous transient outward currents (STOCs) is an important mechanism to regulate arterial myogenic tone, the primary constituent in setting basal vascular tone/resistance [84,85]. K^+^ efflux carried by STOCs causes membrane hyperpolarization and subsequent Ca_V_1.2 closure, resulting in vascular smooth muscle cell relaxation. Estrogen increases the expression of the BK_Ca_ channel β1 subunit and ryanodine receptors in uterine arteries in pregnancy, leading to reduced vascular tone and increased uterine blood flow by promoting Ca^2+^ spark-STOC coupling [80,81,82,83,86]. This adaptation is disrupted by high-altitude hypoxia, resulting in increased uterine vascular resistance [87,88,89]. HIF-1α is a major contributor to the gestational hypoxia-induced uterine vascular maladaptation and HIF-1α-responsive microRNA-210 (miR-210) appears to be a key mediator [55,90,91].

## 3. Mitochondrial Reactive Oxygen Species (ROS): The GOOD, the Bad and the Ugly

### 3.1. Overview of Mitochondrial (ROS)

ROS are reactive molecules derived from molecular O_2_. The major forms of ROS include free radicals superoxide (O_2_^•−^), hydroxyl radicals (OH^•^) and nonradical hydrogen peroxide (H_2_O_2_). ROS are produced from various sources in cells, including NADPH oxidases (NOXs), xanthine oxidase, cytochrome P450 enzymes, endoplasmic reticulum and mitochondria [92,93,94,95]. Among them, the mitochondrion appears to be a major source of ROS under physiological and pathophysiological conditions (see discussion in following sections). ROS in cells are normally maintained at physiological levels, balanced by enzymatic (i.e., superoxide dismutase (SOD), catalase, glutathione peroxidases (GPXs) and peroxiredoxins (PRXs)) and non-enzymatic (i.e., glutathione and vitamins C and E) antioxidant systems. An imbalance between ROS generation and elimination usually cause ROS accumulation, leading to oxidative stress. Oxidative stress contributes to a host of human diseases. As expected, excess ROS appear to be causative factors of uteroplacental dysfunction, contributing to the development of pregnancy complications.

Mitochondria are multifunctional organelles critical to cellular metabolism, Ca^2+^ homeostasis, redox homeostasis, and cell fate. They are the powerhouses of cells and generate ATP via oxidative phosphorylation. Besides ATP, mitochondria also produce precursors for synthesizing marcomolecules such as DNA/RNA, proteins and lipids [96]. In addition, mitochondria are involved in maintaining cellular Ca^2+^ homeostasis [97,98]. Moreover, mitochondria regulate cell death via releasing apoptotic factors such as cytochrome c and caspase activation [99]. ROS are produced as the byproducts of oxidative phosphorylation, participating in regulating cellular redox homeostasis [100].

**Figure 1:**
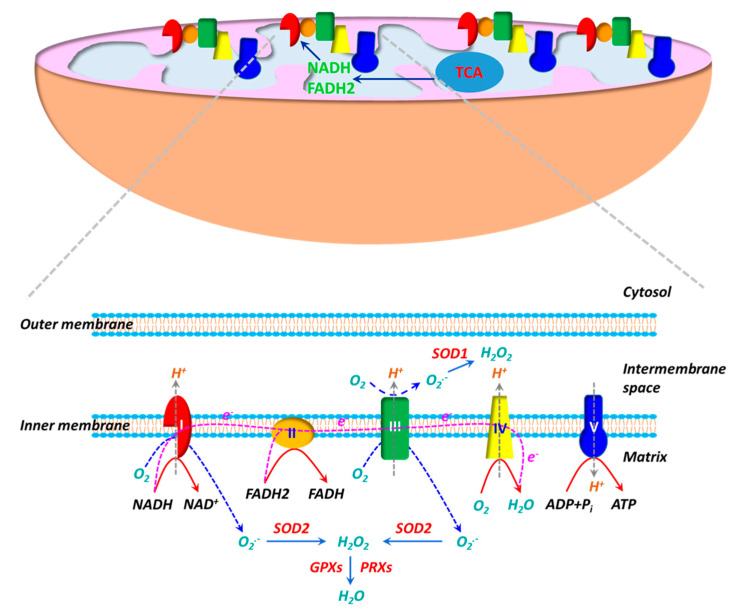
The mitochondrion is the powerhouse in living cells. ATP, the “energy currency”, is synthesized during oxidative phosphorylation. Oxidative phosphorylation is carried out by a series of multi-heteromeric protein complexes (Complexes I-V) of the electron transport chain (ETC) located in the inner mitochondrial membrane. Electrons from NADH and FADH2 produced in tricarboxylic acid (TCA) cycle flow through Complexes I/II to Complex IV, where O_2_ serves as the final electron acceptor to generate H_2_O. During the electron transfer, protons (H^+^) are pumped out of the mitochondrial matrix into the intermembrane space at Complexes I, III and IV, resulting in a proton gradient and a transmembrane electrical potential. The energy stored in proton gradient is used to synthesize ATP from ADP when protons flow back through Complex V (ATP synthase). The mitochondrion is also an important source of reactive oxygen species (ROS). The electron flow in ETC is not perfect and a fraction of electrons are leaked at Complexes I/III, leading to partial reduction of O_2_ to generate superoxide (O_2_^•−^). O_2_^•−^ generated at Complex I is released into the matrix, whereas O_2_^•−^ produced at Complex III is delivered into both the matrix and the intermembrane space. O_2_^•−^ is then dismutated to H_2_O_2_ by superoxide dismutase 1 (SOD1) in the intermembrane space and by SOD2 in the matrix. H_2_O_2_ is subsequently decomposed to H_2_O by glutathione peroxidases (GPXs) and peroxiredoxins (PRXs).

The electron transport chain (ETC), consisting of five multi-subunit protein complexes (Complexes I-V), is located in the inner mitochondrial membrane. Enzymatic reactions in the tricarboxylic acid (TCA) cycle generate reducing equivalents NADH and FADH2, which deposit electrons to the ETC at Complexes I and II, respectively. During the electron flow from NADH or FADH2 to O_2_ through the ETC, protons (H^+^) are pumped from the mitochondrial matrix into the intermembrane space by Complexes I, III and IV. O_2_ acts as an electron acceptor and is reduced to H_2_O at Complex IV. H^+^ ions flow down their gradient and back into the matrix through Complex V (ATP synthase) and the energy stored in the H^+^ gradient is coupled to the synthesis of ATP from ADP and Pi. ROS production is inherent to the oxidative metabolism in mitochondria (Figure 1). Approximate 1–2% of O_2_ reacts with electrons leaked from Complexes I and III during oxidative phosphorylation, leading to partial reduction of O_2_ and generation of superoxide (O_2_^•−^) [101,102,103]. Due to the high rate of O_2_ consumption in mitochondria, a significant fraction of O_2_^•−^ is generated during oxidative phosphorylation. It is believed that mitochondria are the main sources of ROS in cells. O_2_^•−^ generated in Complex I is exclusively released into mitochondrial matrix, whereas O_2_^•−^ produced in Complex III is deposited into both mitochondrial matrix and intermembrane space. O_2_^•−^ is dismutated to hydrogen peroxide (H_2_O_2_) by SOD2 (Mn-SOD) in the matrix and SOD1 (Cu, Zn-SOD) in the intermembrane space [102,104]. H_2_O_2_ can react with Fe^2+^/Cu^+^ via the Fenton reaction to yield hydroxy radical (OH^•^) [105], whereas O_2_^•−^ may react with nitro oxide (NO) to produce peroxynitrite (ONOO^−^)[106]). Both OH^•^ and ONOO^−^ are strong oxidants. Mitochondria have their own antioxidant mechanisms to quench ROS. H_2_O_2_ is primarily decomposed into H_2_O by GPX1/4 and PRX3/5 [104,107].

### 3.2. Hypoxia and Mitochondrial ROS

**Figure 2:**
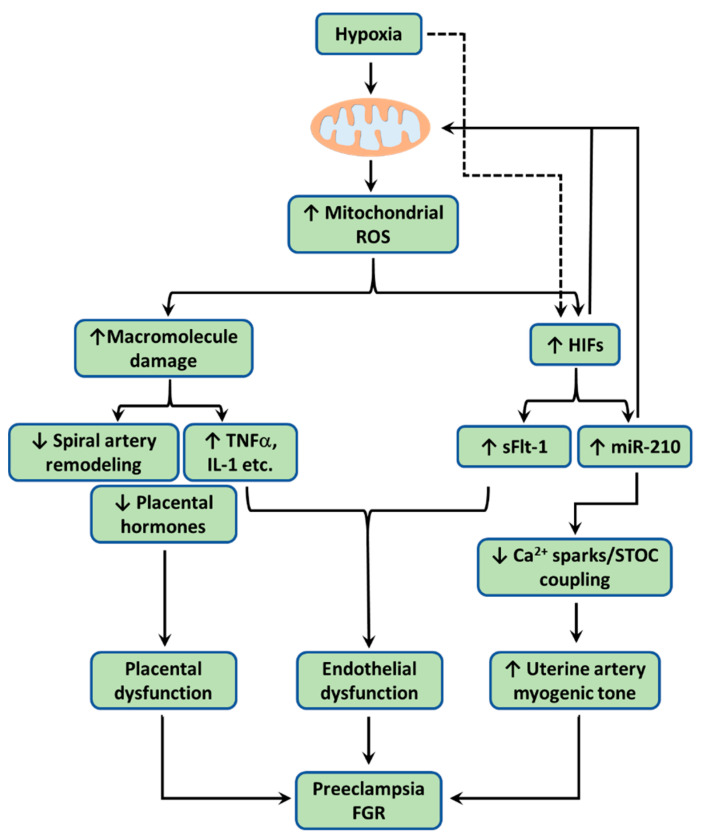
When the uteroplacental cells encounter hypoxia, mitochondria overproduce reactive oxygen species (ROS). Excess ROS cause lipid peroxidation, protein carbonyl, and DNA damage, resulting mitochondrial dysfunction. Dysfunctional mitochondria engender placental dysfunction by (1) promoting trophoblast apoptosis, reducing trophoblast invasion, and consequently impairing spiral artery remodeling, (2) increasing production/release of pro-inflammatory cytokines including TNFα, IL-1β, and IL-6, and (3) suppressing production/release of placental hormones including estrogen, chorionic gonadotropin (CG), and placental lactogen (PL). Hypoxia also via mitochondrial ROS stabilizes hypoxia inducible factors (HIFs), which induces the expression of microRNA-210 (miR-210) and soluble Fms-like tyrosine kinase-1 (sFlt-1). HIF-1 could also in turn reduces mitochondrial metabolism. Once released into the maternal circulation, both pro-inflammatory cytokines and sFlt-1 cause endothelial dysfunction and/or impaired angiogenesis. Pro-inflammatory cytokines and sFlt-1 also act via autocrine/paracrine signaling to disrupt placental function. HIF-responsive miR-210 acts on mitochondria to downregulate iron-sulfur cluster scaffold (ISCU) in the electron transport chain (ETC) to increase mitochondrial ROS. miR-210 suppresses the Ca^2+^ spark/spontaneous transient outward current (STOC) coupling in uterine arteries directly or indirectly via miR-210-induced mitochondrial ROS, leading to increased arterial myogenic tone. Together, these changes conferred by hypoxia-induced mitochondrial ROS contribute to the pathogenesis of preeclampsia and FGR.

The other important function of mitochondria is O_2_ sensing. O_2_ is used as the final electron acceptor in the ETC during oxidative phosphorylation. Mitochondria consume more than 90% cellular O_2_ to generate ATP. Mitochondria are thus extremely sensitive to O_2_ supply and undergo metabolic adaptation when O_2_ deficiency (i.e., hypoxia) occurs. The ETC is proposed to function as an O_2_ sensor [108]. The primary site of ROS production during hypoxia appears to be Complex III [109,110]. In response to hypoxia, ROS are released from Complex III, which in turn stabilize HIFα (Figure 2). This notion has been confirmed with genetic knockout and pharmacological intervention [109,111,112]. Additionally, hypoxia also stimulates ROS release from Complex I, which also contributes to HIF stabilization [113,114]. HIFα is then translocated into the nucleus and dimerises with HIF1β subunit and regulates the expression of genes involved in a diversity of biological processes through binding to the hypoxia response element (HRE). Consequently, HIFs may reduce mitochondrial activity by altering the expression of key enzymes in the TCA cycle and subunits in the ETC. This HIF-mediated adaptation to hypoxia is important for cell’s survival.

### 3.3. Dual Roles of Mitochondrial ROS: Signaling Molecules and Detrimental Effects

Evidently, ROS production is a normal part of cell functions and their level inside the cell is delicately balanced by antioxidants. ROS generated in mitochondria appear to exert their actions in a compartmentalized manner. O_2_^•−^ in the mitochondrial matrix is largely retained to impact mitochondrial function due to its negative charge. However, O_2_^•−^ in the intermembrane space may be transported to the cytosol via voltage-dependent anion channels [115]. On the other hand, nonpolar H_2_O_2_ is able to diffuse from mitochondria into the cytosol to alter other cellular processes. ROS can be either beneficial at low levels or potentially toxic if unchecked. ROS at low levels are signaling molecules to regulate a variety of cellular processes to sustain cellular proliferation, differentiation, angiogenesis, inflammation, and activate stress-responsive survival pathways [116,117,118,119,120]. ROS signaling is predominantly conferred by H_2_O_2_ that exerts the regulatory role through oxidative modification of cysteine residues on target proteins such as enzymes and transcription factors [117]. The oxidation of the cysteine thiol (Cys-SH) to the sulfenic form (Cys-SOH) results in allosteric changes in the protein leading to alternation of its function. In addition, ROS also possess antimicrobial action and participate in regulating autophagy [118,121,122].

However, ROS are highly reactive and their overproduction can cause oxidative damage to macromolecules such as lipids, proteins and nucleic acids and even elicits cell apoptotic cell death. Lipid peroxidation often occurs as the oxidative damage of polyunsaturated fatty acids due to the attack of carbon-carbon double bond(s) by ROS, leading to impairment of membrane function [123]. Malondialdehyde (MDA) and 4-hydroxynonenal (4-HNE) are two main lipid peroxidation products. Protein oxidation often leads to irreversibly forming a reactive carbonyl moiety in a protein, such as an aldehyde and ketone. Protein carbonylation causes protein misfolding and/or altering protein conformation, leading to protein inactivation [124]. ROS also cause DNA damage by causing base lesions and double strand breaks, which are potentially mutagenic [125]. 8-Hydroxydeoxyguanosine (8-oxodG) is one of the common products of ROS-induced DNA damage [126]. Overall, these ROS modifications would disrupt membrane integrity, alter chromatin structure/gene expression, and inactivate protein function, leading to cellular dysfunction and even cell death. For example, excess O_2_^•−^ has been shown to inactivate mitochondrial aconitase by disrupting the iron–sulfur cluster [127], whereas H_2_O_2_ could induce mitochondrial lipid peroxidation [128]. Mitochondria DNAs are susceptible to oxidative damage and the resultant mutations in turn alter ETC activity and escalate ROS production [129]. Therefore, mitochondrial structure and function could be impaired by ROS overproduction in mitochondria.

## 4. Mitochondrial ROS and Preeclampsia/FGR

### 4.1. Mitochondrial ROS Production in Preeclamptic/FGR Uteroplacental Tissues

Normal pregnancy is characterized by mild oxidative stress, which is exaggerated in preeclampsia and FGR [130]. Numerous studies reveal that uteroplacental ROS production is increased, inferenced directly from the measurement of O_2_^•−^/H_2_O_2_ or indirectly from the measurement of markers of oxidative stress (i.e., MDA, 4-hydroxynonenal, 8-isoprostane) [90,131,132,133,134,135,136,137,138,139,140,141]. On the other hand, the enzymatic antioxidant system in uteroplacental tissues/cells appears to be impaired in preeclampsia and FGR. The expression and/or activity of SODs, GPXs, thioredoxin reductases (TRXRs), and catalase are reduced in preeclamptic and FGR placentas and trophoblasts [132,133,134,135,136,142,143,144,145,146,147]. Evidently, these changes engender overwhelming ROS accumulation in uteroplacental cells, leading to heightened oxidative stress. Abundance of SOD1 and catalase in preeclamptic myometrium is similarly decreased [148].

The association of mitochondrial dysfunction with preeclampsia is first reported in 1989 [149]. In this study, a high incidence of pre-eclampsia in a family with mitochondrial dysfunction is observed. Since then, emerging evidence has implicated mitochondrial dysfunction in the etiology of pregnancy complications [150,151]. Placental oxidative phosphorylation capacity is reduced in preeclampsia [152]. Comparative proteomic analysis reveal that various proteins involved in oxidative phosphorylation/ETC are altered in preeclamptic placentas [153,154]. Various ETC complexes as well as their subunits such as Complexes I-IV, cytochrome c oxidase (COX), are found to be downregulated in both preeclamptic and FGR placentas [155,156,157,158]. Accordingly, activity of one or more of ETC complexes in the placenta is suppressed in preeclampsia and FGR [157,158,159,160,161]. However, the work from Cetin’s laboratory demonstrates that cytotrophoblast ETC activity is increased in placentas of FGR [162]. Animal models of preeclampsia in rats and FGR in pigs also show downregulation of placental ETC components and activities [163,164,165]. The reduced expression and activities of placental ETC complexes are associated with increased mitochondrial H_2_O_2_ production [163]. Placental insufficiency is common feature shared by preeclampsia and preterm birth [166]. Preeclampsia is also a major contributor to preterm birth [167]. Placenta from preterm birth displays reduced mitochondrial calcium uptake 1 (MICU1) [168], a component of mitochondrial calcium uniporter (MCU) that participates in regulating mitochondrial Ca^2+^ homeostasis. MICU1 functions as a gatekeeper to prevent mitochondrial Ca^2+^ overload and its deficiency leads to Ca^2+^ overload and subsequent ROS overproduction and apoptosis [169]. 

Detection of oxidative modifications of intracellular molecules is frequently used as a marker of ROS formation. The increase in placental mitochondrial ROS in preeclampsia and FGR is often inferred from the observations of escalated lipid peroxidation in mitochondria in early studies. For example, Wang and Walsh observed that levels of MDA are elevated in mitochondria isolated from preeclamptic placentas [131]. Shibata and colleagues detected accumulation of 4-HNE-modified proteins in both cytosol and mitochondria with a dominance in mitochondria [170]. The development of fluorescent probes has enabled monitoring ROS generation in cells and even in mitochondria. For instance, the increase in mitochondrial O_2_^•−^ in the preeclamptic placenta is confirmed using MitoSOX Red [171]. A similar increase in mitochondrial H_2_O_2_ is also detected using Amplex red in the placentas of reduced uterine perfusion pressure (RUPP) rat model of preeclampsia [163]. Intriguingly, Holland et al. reported that H_2_O_2_ (detected with Amplex Ultra Red) production and antioxidant activity were increased in term preeclamptic placentas but decreased in pre-term preeclamptic placentas [141]. 2′,7′-Dichlorodihydrofluorescein diacetate (H2DCFDA) is commonly used for measuring cellular H_2_O_2_ [172]. Increased ROS in preeclamptic placentas are detected with H2DCFDA [157]. Using this probe in the absence or presence of the mitochondrial targeted antioxidant mitoQ, it is determined that the increased cellular H_2_O_2_ is predominantly from mitochondria in both labyrinth and junctional zones of the placenta from a rat model of gestational hypoxia [173]. The increase in mitochondrial O_2_^•−^ in primary trophoblasts in response to chronic hypoxia is also detected using MitoSOX Red [171]. 

### 4.2. Uteroplacental Tissues Exhibit Oxidative Stress due to Hypoxia-Induced Mitochondrial Dysfunction in Preeclampsia/FGR

Hypoxia is a common feature of preeclampsia and FGR [17,174]. Placental hypoxia is long believed to induce oxidative stress by stimulating ROS generation in mitochondria and other compartments of uteroplacental cells, leading to placental dysfunction and subsequent development of preeclampsia and FGR [17,18,174]. The placenta is a metabolically active organ and has a high nutrient and energy demand. Oxidative phosphorylation in the placenta increases progressively throughout normal pregnancy [175]. Unsurprisingly, the placenta at term consumed ~60–70% glucose and ~50% of O_2_ supplied by the uteroplacental circulation [176,177]. ATP generated by mitochondria fuels placental functions, including placental growth, protein and hormone synthesis and active transport, which are essential for placental/fetal growth and development. The placental function is disrupted by hypoxia [178]. The high-rate consumption of O_2_ makes mitochondria to be the major source of ROS in placental cells.

The link between hypoxia and oxidative stress in uteroplacental tissues/cells has been established based on both in vitro and in vivo studies. Human placentas from high-altitude pregnancy exhibit decreased activities of SODs, GPXs, and TXRs along with increased levels of lipid peroxidation, protein carbonyl, and nitrotyrosine [179]. In first trimester placental explants, the expression of SOD1 and SOD2 is reduced by hypoxia [180]. Uterine arteries from pregnant sheep at high altitude also have elevated ROS [90]. In a rat model of hypoxic pregnancy, an increase in 4-HNE is observed in the placenta [181]. Gestational hypoxia also increases peroxynitrite formation in placentas of guineapigs [182] and catechol-O-methyltransferase-deficient (COMT^−/−^) in mice [183]. Exposure of BeWo trophoblastic cells to hypoxia elevates intracellular ROS levels and mitoQ largely prevents hypoxia-induced ROS increase [184]. This finding is corroborated by Aljunaidy and colleagues who demonstrate that gestational hypoxia-induced increase in placental ROS in rat is blocked by mitoQ [173]. These observations suggest a predominant mitochondrial contribution to the accumulation of cytosolic ROS. Unsurprisingly, mitochondrial-derived superoxide production in human primary trophoblasts is increased after hypoxia exposure [171]. Similarly, in vitro hypoxia/reoxygenation also increases mitochondrial H_2_O_2_ production in healthy term placenta [141].

Mitochondrial proteins and enzyme activities are often used as markers of mitochondrial content. Hypoxia is also found to impact mitochondrial content in the uteroplacental unit. Compared to sea-level pregnancy, high-altitude pregnancy reduces Complex I level in human placenta [178]. Mitochondrial content is reduced in both placental explants and cultured BeWo cells following hypoxia exposure [184]. Activities of Complexes I and/or IV of the ETC are reduced in the hypoxic guinea pig placentas [182] and in hypoxic rat placentas [185]. Correspondingly, Complex I- and Complex-IV-mediated respiration is repressed by hypoxia in cultured trophoblast-like JEG3 cells [178]. The HIF-responsive miR-210 appears to be a major mediator of hypoxia-induced changes in the ETC. The expression of uteroplacental miR-210 is upregulated in preeclampsia, FGR and high-altitude pregnancy [55,178,186,187,188,189]. MiR-210 transfection suppresses the expression of iron-sulfur cluster scaffold (ISCU), succinate dehydrogenase (SDHD), NDUFA4, and cytochrome c oxidase assembly protein (COX10) in trophoblasts [178,190]. Any alternation of ETC complexes could potentially influence ETC flow and mitochondria-mediated ROS production. Trophoblasts transfected with miR-210 have reduced mitochondrial respiration [157,190]. MiR-210-induced downregulation of ISCU disrupts the electron flow within Complex I and reduces its activity, leading to increased ROS production [191,192].

### 4.3. Mitochondrial ROS Overproduction and Uteroplacental Dysfunction in Preeclampsia/FGR

#### 4.3.1. Mitochondrial ROS Leads to Mitochondrial Dysfunction

The excess ROS in preeclamptic/FGR placentas cause damage to macromolecules as evidenced by: (1) increased indices of lipid peroxidation such as MDA and 4-HNE [131,135,143,193,194], (2) elevated levels of protein carbonyl [143,195], and (3) raised levels of DNA damage markers such as 8-OHdG and γH2AX and increased DNA fragmentation [196,197,198,199] (Figure 2). The DNA damage could be imitated by exposing decidual stromal cells to H_2_O_2_ or hypoxia/reperfusion [199] and in animal models of preeclampsia and FGR [164,200,201]. Mitochondria are susceptible to damage by ROS, resulting in altered structure and function. Mitochondrial dysfunction promotes oxidative stress and the overproduction of ROS in turn could cause further mitochondrial damage, form a vicious cycle [202,203]. Lipid peroxidation appears to primarily occur in mitochondria of preeclamptic placentas [170]. Mitochondrial swelling, a hallmark of mitochondrial dysfunction, is frequently detected in preeclamptic/FGR placentas/trophoblasts [147,153,154,157]. Similar findings are also observed in animal models of preeclampsia and FGR [147,164]. Swollen mitochondria are usually associated with irregular cristae (i.e., folds in the inner membrane of a mitochondrion) [154,157]. The complexes of ETC are embedded in the inner membranes of mitochondria. The disruption of cristae would perturb the structure/function of the ETC. Indeed, mitochondria overproduce ROS once they become swollen [204]. There are also increased mutations in the trophoblast mitochondrial genome observed in African American women with preeclampsia [205], probably due to ROS-induced DNA damage. Vishnyakova and colleagues detected both increased mitochondrial DNA copy number and mitochondrial Complex I activity in preeclamptic placentas [206]. The authors speculate that the high respiration rate in Complex I may contributes to the increased mitochondrial ROS production in preeclamptic placentas. Intriguingly, mitochondrial DNA copy number could be regulated by mitochondrial ROS. H_2_O_2_ exposure increases yeast mitochondrial DNA copy number [207]. Moreover, mitochondrial DNA copy number is positively associated with increased oxidative stress in placental tissues from gestational diabetes [208].

#### 4.3.2. Mitochondrial ROS Impair Trophoblast Invasion/Spiral Artery Remodeling

As aforementioned, the failure of trophoblast invasion/spiral artery remodeling results in placental dysfunction and is the major contributor to the development of preeclampsia and FGR [33,209]. Mounting evidence from the human and animal models of preeclampsia and FGR implicates hypoxia in the impaired trophoblast invasion and defective spiral artery remodeling [57,61,67,68,210,211] (Figure 2). The inhibition of invasion by hypoxia has been imitated in vitro using primary trophoblasts and trophoblast cell lines [212,213]. In the cultured human extravillous trophoblast cell line HTR-8/SVneo, hypoxic treatment increased mitochondrial ROS and decreased invasive ability [214]. Importantly, H_2_O_2_ exposure decreased the invasion of human trophoblast cell-lines HTR-8/SVneo and TCL-1 [215,216]. Trophoblast apoptosis is commonly observed in preeclampsia and FGR [217,218,219]. The invasion of trophoblast is greatly impacted by trophoblast apoptosis [220]. Increased trophoblast apoptosis could limit trophoblast invasion. Hypoxia enhances trophoblastic apoptosis in cultured villous tissues and primary trophoblasts [221,222]. Hypoxia/reoxygenation-induced mitochondrial cytochrome c release and apoptosis in trophoblasts of the cultured villous tissue could be diminished by the free radical scavenger, desferrioxamine [223]. Similarly, hypoxia also stimulates H_2_O_2_ production in HTR-8/SVneo cells and promotes apoptosis [224]. The hypoxia-induced apoptosis is reduced by catalase. These observations suggest a potential role of ROS in trophoblast apoptosis induced by hypoxia. This notion is corroborated by the observations that H_2_O_2_ directly induces apoptosis or reduces viability in trophoblast cell lines [216,225,226]. Moreover, mitochondrial ROS stimulated by rotenone is found to promote trophoblast apoptosis [227]. Mitochondria-derived ROS stabilizes HIF-1α. HIF-1α appears to be the principal mediator of comprised spiral artery remodeling as discussed in previous sections. Specific overexpression of HIF-1α in trophoblasts disrupts trophoblast invasion and results in failure to remodel spiral arteries [68]. On the other hand, knockdown of HIF-1α reduces hypoxia-induced apoptosis of HTR8/SVneo cells [65]. As discussed previously, HIF-1-responsive miR-210 in the placenta is elevated in preeclampsia, FGR and high-altitude pregnancy and inhibits both trophoblast invasion and uterine vascular adaptation [55,178,186,187,188,189,213]. MiR-210 targets and downregulates ISCU, NDUFA4, and SDHD of the ETC, leading to reduced mitochondrial respiration and increased ROS production [188,190,191,192] (Figure 2). As expected, miR-210-induced mitochondrial dysfunction contributes to the impaired trophoblast invasion [190].

#### 4.3.3. Mitochondrial ROS Impair Placental Hormone Production/Secretion 

The placenta secrets a variety of hormones to regulate placental growth and transport as well as maternal physiology, which are essential for fetal growth/development [228,229,230]. Accumulating evidence reveals that the production/secretion of various placental hormones is impaired by hypoxia via ROS (Figure 2). Estrogen is primarily synthesized and secreted by the placenta during pregnancy and is essential for the adaptive changes of maternal cardiovascular function during pregnancy including hemodynamic adaptation of the uteroplacental circulation, placental cell differentiation, proliferation and angiogenesis [231,232]. Compared to normal pregnancy, circulating estrogen level is reduced in preeclamptic and FGR pregnancy [233,234,235]. High altitude pregnancy also has less circulating estrogen than sea-level pregnancy [236,237]. As expected, estrogen production in preeclamptic placental explants is suppressed compared to normotensive term counterparts [238]. Intriguingly, the reduction of estrogen synthesis in preeclamptic placentas is replicated by treating first trimester placental explants with H_2_O_2_ [238]. The reduced estrogen production is probably due to H_2_O_2_-mediated inhibition of aromatase activity [239]. Human chorionic gonadotropin (hCG) produced by the trophoblasts participates in regulating placental development, trophoblast invasion, and angiogenesis/vasculogenesis [240]. Altered hCG is a marker of preeclampsia and FGR [241,242]. Low hCG in the late first trimester is associated with FGR [242]. Hypoxia downregulates hCG expression/production in JEG-3, BeWo, and JAr cells [243]. The reduction of hCG by hypoxia could be simulated by exposing JEG-3 cells to H_2_O_2_ [244], suggesting that ROS probably mediate the hypoxia-induced downregulation of hCG in the placenta. Human placental lactogen (hPL) is also produced by trophoblasts and promotes placental/fetal growth primarily through insulin-like growth factors (IGFs) [245,246]. Deletion of the P0 transcript of IGF2 gene results in reduced placental growth and FGR [247]. Interestingly, increasing mitochondrial ROS by inhibiting Complex I activity with rotenone reduced hPL and IGF2 expression [248], implicating a pivotal role of mitochondrial ROS in the etiology of FGR.

#### 4.3.4. Mitochondrial ROS Promote Placental Release of sFlt-1 

The trophoblastic production and release of the antiangiogenic factor sFlt-1 are increased in preeclampsia and FGR [71,201,249,250]. The release of sFlt-1 into the maternal circulation is responsible for inducing proteinuria and hypertension by causing endothelial dysfunction [70,251,252,253]. Apparently, sFlt-1 release is mainly induced by hypoxia as sFlt-1 levels in placentas from high-altitude pregnancy are higher than placentas from sea-level pregnancy [69]. This is confirmed in a rat model of chronic hypoxia [254] and in vitro studies using placenta explants and trophoblasts [69,201,255,256,257]. Concurrently, exposure to hypoxia also increases oxidative stress in placental tissues and trophoblasts [255,256,257] (Figure 2). Significantly, H_2_O_2_ promotes sFlt-1expression in placental villous explants and BeWo cells [201]. Furthermore, the concomitant increases in mitochondrial ROS and sFlt-1 production are blocked by the mitochondria-targeted hydrogen sulfide donor AP39, thus establishing mitochondrial ROS as the link between hypoxia and sFlt-1 release [171]. Intriguingly, sFlt-1 also exerts its autocrine/paracrine effects on the placenta. The administration of sFlt-1 into pregnant mice increased oxidative stress in placentas, causing mitochondrial swelling in trophoblasts and apoptosis [258,259]. Similarly, induction of human sFlt-1 in pregnant mice impairs placental vascularization and nutrient exchange function, leading to FGR [260].

#### 4.3.5. Mitochondrial ROS Promote Placental Release of Cytokines 

Trophoblasts and immune cells in the placenta produce cytokines. Preeclampsia/FGR is commonly associated with increased expression/production of pro-inflammatory cytokines such as TNF-α, IL-1α/β, IL-6 and IL-8 and decreased expression/production of anti-inflammatory cytokine IL-10 in the placenta [261,262,263,264,265,266,267]. The altered expression/production of placental cytokines is believed to be the consequence of hypoxia [268]. Indeed, hypoxia or hypoxia/reoxygenation increases the expression/production of TNF-α, IL-1α, IL-1α, IL-6, and IL-8 in cultured placental explants and BeWo cells [184,269,270] (Figure 2). Expectedly, H_2_O_2_ stimulates TNF-α and IL-1β production and reduces IL-10 generation in placental explants [271]. In addition, hypoxia-induced increase in pro-inflammatory cytokine expression is associated with elevated mitochondrial ROS in BeWo cells [184]. The inhibition of mitochondrial ROS by diphenyleneiodonium diminishes lipopolysaccharide-induced cytokine production in human immune cells harboring TNF receptor 1 mutations [272]. In a way similar to sFlT-1, pro-inflammatory cytokines cause endothelial dysfunction once released into the maternal circulation from the placenta [273,274]. Locally, TNF-α inhibits trophoblast fusion and hCG-β expression [275]. An in vitro study reveals that TNF-α and IL-6 also increase trophoblast apoptosis, resulting in increased trophoblasts shed from the placenta [276].

#### 4.3.6. Mitochondrial ROS Impair Uterine Vascular Adaptation

Pregnancy is associated with dramatically reduced uterine vascular resistance and increased uterine blood flow in both human and experimental animals [277,278,279,280,281,282,283]. In addition to structural remodeling of uterine arteries, functional changes in the uterine arteries also contributes to the uterine vascular adaptation [14,232,284]. However, gestational hypoxia thwarts the adaptation of the uteroplacental circulation. Uterine blood flow is reduced and uterine vascular resistance is increased in preeclamptic, FGR, and high-altitude pregnancy and in animal models of chronic hypoxia [28,31,32,55,60,62,285,286,287]. Mechanistically, the maladaptation of the uteroplacental circulation is in part due to the impaired Ca^2+^ spark/STOC coupling conferred by hypoxia-induced upregulation of miR-210 and ROS overproduction in uterine arteries [55,89,90,91,288,289] (Figure 2). Given the concomitant increase of both miR-210 and oxidative stress in uterine arteries in response to gestational hypoxia and the role of miR-210 in promoting mitochondrial ROS, it is expected that mitochondrial ROS in uterine arteries probably contributes to the dysfunction of uterine arteries in pregnancy complications associated with hypoxia, which merits further investigation.

## 5. Potential Interventions to Prevent/Manage Preeclampsia/FGR

As aforementioned, oxidative stress is a leading contributor to the pathogenesis of preeclampsia/FGR. It is expected that restoring redox homeostasis with the therapeutic approach of antioxidants would be effective for the treatment of both complications. Despite of success in preclinical studies, the antioxidant therapy for preeclampsia/FGR has proved largely unsuccessful in clinical trials. For example, the supplement with vitamins C and E does not reduce the incidence of both disorders and sometimes even results in deleterious outcomes [290,291,292]. Many factors could contribute to the failure of antioxidant therapy for the treatment of preeclampsia/FGR in clinical trials. Among them are poor bioavailability of antioxidants in tissues/cells undergoing oxidative stress and sites of ROS production in the cells and indiscriminate abolishment of ROS including those are essential for physiological signaling. These findings require to develop optimal antioxidant therapies “selectively inhibiting only the disease-relevant ROS sources and leaving all others intact” [293]. Placental mitochondria-targeted antioxidants may alleviate oxidative stress without suppressing ROS from non-mitochondrial sources that are indispensable to physiological signaling and ROS-mediated cellular functions in other maternal and fetal tissues. 

### 5.1. Interventions Selectively Target Placental Cells

Preeclampsia and FGR are most common pregnancy complications with high morbidity and mortality for both the mother and fetus. Although our understanding of the pathophysiology of both disorders has advanced significantly, there are still no effective cures available. The only applicable treatment for both preeclampsia and FGR is either delaying labor or inducing labor. Therefore, there is a great need to develop effective therapy for preeclampsia and FGR. Given that both preeclampsia and FGR are originated from the uteroplacental dysfunction, it is anticipated that improving uteroplacental function is a promising therapeutic approach. To this end, it is important to specifically deliver drugs to the placenta to minimize both side effects in other maternal tissues and fetal exposure. Recent studies reveal that targeting ligands can be selectively delivered to the placenta in mice by conjugating with various peptides which bind to surface receptors in placental cells. Tumor-homing peptide sequences CGKRK and iRGD are found to bind to human and mouse placental tissues [294]. These peptides facilitate targeted delivery of peptide-decorated liposomes containing IGF2 to the mouse placenta, improving fetal weight in the P0 mouse model of FGR [294]. Moreover, the synthetic peptide CNKGLRNK is found to selectively bind to the endothelium of the uterine spiral arteries and placental labyrinth in mice and administration of CNKGLRNK-decorated liposomes containing nitric oxide donor 2-[[4-[(nitrooxy)methyl]benzoyl]thio]-benzoic acid methyl ester (SE175) reduces placental 4-HNE, increases mean spiral artery diameter and fetal weight [295]. Similarly, an alternative placental-specific drug delivery system is developed by Zhang and colleagues using synthetic placental chondroitin sulfate A-binding peptide (CSA-BP) based on the finding that Plasmodium falciparum-infected erythrocytes bind to CSA on the surfaces of trophoblasts [296]. Following intravenous administration, CSA-BP-conjugated nanoparticles accumulate in the mouse placenta without detection in fetal tissues [296]. Encouragingly, placental CSA-BP conjugated to lipid-polymer nanoparticles containing sFlt-1 siRNAs effectively reduces placental and serum sFlt-1 levels in mice [297]. 

### 5.2. Interventions Selecetively Target Mitochondrial ROS

As discussed in previous sections, studies over last decades have implicated that hypoxia-induced mitochondrial ROS production is a major contributor to placental dysfunction and subsequent development of preeclampsia and FGR. Thus, relieving mitochondria-associated oxidative stress to restore placental function presents a promising strategy for treating these disorders. This therapeutic potential is auspiciously supported by findings from numerous in vitro and in vivo studies as well as animal models. A number of antioxidants have been found to effectively improve mitochondrial ROS-induced placental dysfunction. In this section, we focus the discussion on potential antioxidant therapies of selenium, melatonin, and mitochondria-targeted MitoQ in preeclampsia and FGR.

#### 5.2.1. Selenium

Selenium, an essential trace element, participates in regulating cell functions in the form of selenocysteine which is incorporated into in selenoproteins including antioxidant enzymes GPXs and TXRs, two major antioxidant enzymes in mitochondria. Disrupting selenocysteine incorporation into selenoproteins by knock-downing selenocysteine insertion binding protein 2 (SECISBP2) in trophoblast cell lines JAR and JEG-3 cells leads to elevated oxidative stress, impaired trophoblast invasion, and reduced the expression of hCGβ [298]. Selenium deficiency is associated with preeclampsia and FGR [299]. In rat models, selenium deprivation reproduces preeclampsia-like symptoms and FGR, accompanied with reduced activities of placental GPXs and TXRs and increased lipid peroxides/protein carbonyls in the placenta [300,301]. In BeWo, JEG-3 and Swan-71, selenium supplement increases the expression/activities of GPXs and TXRs and effectively diminishes rotenone- and antimycin-induced mitochondrial oxidative stress [227,302]. In small-scale clinical trials, selenium supplement is found to decrease the incidence of preeclampsia [303,304] and decreases the circulating level of sFlt-1 [305]. A recent large-scale trial conducted in Norway demonstrates that higher maternal dietary selenium intake is significantly associated with a lower risk of FGR [306].

#### 5.2.2. Melatonin 

It is well-known that melatonin secreted by the pineal gland participates in regulating circadian rhythm. Interestingly, melatonin possesses antioxidant property by directly scavenging ROS and indirectly promote the expression/activity of antioxidant enzymes [307,308]. Melatonin is also synthesized and its receptors are expressed in human placentas [309,310,311], implicating its autocrine/paracrine effects in regulating placental function. The expression of aralkylamine N-acetyltransferase, a key enzyme in melatonin synthesis, and melatonin receptors 1 and 2 in the placenta is reduced in preeclampsia and FGR [312,313]. Melatonin is believed to enter mitochondria through peptide transporters PEPT1 and PEPT2 [314], which enables melatonin to function as an antioxidant to counter overproduction of mitochondrial ROS-induced placental dysfunction in preeclampsia and FGR. Melatonin prevented hypoxia/reoxygenation-induced ROS, apoptosis, autophagy, and inflammation in cultured human primary villous trophoblasts [315,316,317]. Melatonin also increases antioxidant defense by upregulating the expression of glutamate cysteine ligase (the first and rate-limiting enzymes in the production of the cellular antioxidant glutathione (GSH)), NADPH: quinone acceptor oxidoreductase 1 and thioredoxin in placental tissues [318]. Moreover, melatonin suppresses sFlt-1 secretion in primary trophoblasts [318]. In the RUPP rat model, melatonin lowers mean arterial pressure and placental expression of sFlt-1 [319]. Both human and animal studies prove beneficial effects of melatonin in treating preeclampsia and FGR. Maternal administration of melatonin improves ischemia/reperfusion-induced oxidative DNA and impaired mitochondrial respiration in the placenta, leading to improved fetal growth [200,320]. Promisingly, melatonin treatment significantly prolonged the interval from diagnosis to delivery in women with preeclampsia [321]. However, antenatal treatment with melatonin during the last one-third of gestation is found to decrease birth weight in high-altitude pregnant sheep [322], suggesting existence of species-dependent melatonin effects.

#### 5.2.3. mitoQ

A number of mitochondria-target antioxidants have been developed by conjugating the lipophilic triphenylphosphonium (TPP^+^) cation to antioxidant moieties [323,324]. Due to their positive charges, these compounds can easily pass biological membranes and accumulate several-hundred-fold within mitochondria. Therefore, they are able to protect against mitochondrial oxidative damage. Among them, mitoQ, consisting of a ubiquinone moiety attached to TPP^+^, has shown promise in animal models of cardiovascular diseases and in a clinical trial [325,326,327]. The potential therapeutics for preeclampsia and FGR with mitoQ has also been intensively investigated. Following administration, mitoQ is found to accumulate in rat and sheep placentas [328,329]. In a rat model of prenatal hypoxia, treatment with mitoQ prevents hypoxia-induced decrease in mitochondrial activity and increase in placental ROS [173,185,328,330,331]. In both rat and sheep, impaired fetal growth is improved by mitoQ in hypoxic pregnancy [328,329]. Similarly, mitoQ also improves ETC activities, reduces mean arterial pressure, and mends fetal growth in a rat RUPP model [163]. Interestingly, in a mouse RUPP model, mitoQ administration during late pregnancy (GD 13.5–17.5) reduces placental oxidative stress, lowers blood pressure, and improves fetal birth weight, whereas mitoQ application during early pregnancy (GD 7.5–11.5) exacerbates RUPP-induced preeclampsia-like symptoms [147]. Therefore, timing is an important factor to be considered when administrating antioxidants during pregnancy as mitochondrial ROS could participate in regulating placental development in early pregnancy [147,332]. In a way similar to mitoQ, AP39, consisting of a H_2_S-donating moiety (dithiolethione) coupled to TPP^+^, also prevents ROS production, lowers HIF-1α, and decreases sFlt-1 production in cultured human primary trophoblast culture [171].

## 6. Conclusions

Placental homeostasis is essential for the well-beings of the mother and normal growth of the fetus. Mitochondria play a central role in maintaining placental homeostasis. Mitochondrial function is sensitive to changes in O_2_. Although a low O_2_ environment is indispensable for embryonic development and placentation, prolonged hypoxia is detrimental to the placenta as it promotes mitochondrial dysfunction and ROS overproduction. Numerous studies demonstrate that excessive ROS cause damages to lipids, proteins and DNAs, resulting in impaired trophoblast invasion/spiral artery remodeling, reduced hormone production/secretion, and increased release of bioactive factors into the maternal circulation. Together, these changes contribute to placental dysfunction and subsequent development of preeclampsia and FGR. Ultimately, targeting mitochondrial ROS may offer avenues for the development of therapeutics. Indeed, promising preclinical results have been obtained in animal models with mitochondria-targeted antioxidants. However, more efforts will be needed for the successful translation of these scientific findings into clinical applications for preeclampsia and FGR. It should also bear in mind that ROS possess both beneficial and detrimental roles in human body. It will be another great challenge to preserve physiological signaling of ROS while alleviating pathological ROS damage.
